# Modulation of apoptosis and Inflammasome activation in chondrocytes: co-regulatory role of Chlorogenic acid

**DOI:** 10.1186/s12964-023-01377-w

**Published:** 2024-01-02

**Authors:** Muhammad Fakhar-e-Alam Kulyar, Quan Mo, Wangyuan Yao, Yan Li, Shah Nawaz, Kyein San Loon, Ahmed Ezzat Ahmed, Aiman A. Alsaegh, Khalid M. Al Syaad, Muhammad Akhtar, Zeeshan Ahmad Bhutta, Jiakui Li, Desheng Qi

**Affiliations:** 1 College of Veterinary Medicine, Huazhong Agricultural University, Wuhan, 430070 People’s Republic of China; 2 Department of Animal Nutrition and Feed Science, College of Animal Science and Technology, Huazhong Agricultural University, Wuhan, 430070 China; 3 Department of Microbiology and Plant Pathology, University of California-Riverside, Riverside, CA 92521 USA; 4 Biology Department, College of Science, King Khalid University, Abha, 61413 Saudi Arabia; 5 Department of Laboratory Medicine, Faculty of Applied Medical Sciences, Umm Al-Qura University, Mecca, Saudi Arabia; 6 Laboratory of Veterinary Immunology and Biochemistry, College of Veterinary Medicine, Chungbuk National University, Cheongju, 28644 Republic of Korea

**Keywords:** Apoptosis, B-cell lymphoma 2, Bcl-2, Interleukin-1β, Inflammasome

## Abstract

**Background:**

The B-cell lymphoma 2 (Bcl-2) protein regulates programmed cell death throughout the disease conditions by upholding apoptotic pathways. However, the mechanism by which it’s expressed in chondrocytes still needs to be studied in chondrocyte-related disorders. Additionally, exploring the potential therapeutic role of Chlorogenic acid (CGA) in confluence with Bcl-2 modulation is of significant interest.

**Methods:**

In vivo and in vitro studies were performed according to our previous methodologies. The chondrocytes were cultured in specific growth media under standard conditions after expression verification of different microRNAs through high-throughput sequencing and verification of Bcl-2 involvement in tibial growth plates. The effect of Bcl-2 expression was investigated by transfecting chondrocytes with miR-460a, siRNA, and their negative controls alone or in combination with CGA. The RNA was extracted and subjected to a reverse transcription-quantitative polymerase chain reaction (RT-qPCR). Western blot analysis and immunofluorescence assays were performed to visualize the intracellular localization of Bcl-2 and associated proteins related to apoptotic and inflammasome pathways. Moreover, apoptosis through flow cytometry was also performed to understand the modulation of concerning pathways.

**Results:**

The suppression of Bcl-2 induced higher apoptosis and mitochondrial dysfunction, leading to IL-1β maturation and affecting the inflammasome during chondrocyte proliferation. Conversely, overexpression attenuated the activation, as evidenced by reduced caspase activity and IL-1β maturation. In parallel, CGA successfully reduced siRNA-induced apoptosis by decreasing Cytochrome C (Cyto C) release from the mitochondria to the cytoplasm, which in turn decreased Caspase-3 and Caspase-7 cleavage with Bcl-2-associated X protein (Bax). Furthermore, siBcl-2 transfection and CGA therapy increased chondrocyte proliferation and survival. The CGA also showed a promising approach to maintaining chondrocyte viability by inhibiting siRNA-induced apoptosis.

**Conclusions:**

Targeting Bcl-2-mediated regulation might be a possible treatment for chondrocyte-related conditions. Moreover, these results add knowledge of the complicated processes underlying chondrocyte function and the pathophysiology of related diseases, highlighting the significance of target specific therapies.

Video Abstract

**Supplementary Information:**

The online version contains supplementary material available at 10.1186/s12964-023-01377-w.

## Introduction

Chondrocytes are highly specialized cells that maintain and repair cartilage tissue. The appropriate activity of chondrocytes is critical for sustaining joint function and general health [1]. The pathophysiology of chondrocyte-related diseases such as rheumatoid arthritis and osteoarthritis is mostly influenced by imbalanced chondrocytes' apoptosis [2, 3]. Therefore, understanding the role of chondrocyte-related apoptosis proteins is vital for developing new therapeutic strategies. Tibial dyschondroplasia (TD) is characterized by producing an unmineralized cartilage core in the tibial growth plate [4]. Lameness and stunted growth are common outcomes of TD, making it a significant economic burden for the poultry industry. The disease is probably be caused by thiram, a common pesticide in lower-income countries [5]. The precise pathological processes in TD are unknown; however, it is associated with inflammation and immunological dysregulation, according to some researchers. The inflammasome is a multiprotein complex that plays a vital role in innate immunity and inflammation. It comprises several proteins, including sensor, adaptor, and effector proteins [6]. When the inflammasome is activated, caspase-1 cleaves and activates proinflammatory cytokines such as interleukin-1β (IL-1β) and interleukin-18 (IL-18), that further stimulate inflammation and immunological responses [7]. The implications of this phenomena for maintenance and function are substantial, particularly in regions with limited potential for regeneration, such as growth plates [8]. Some hypotheses impute to the existence of multiple origins, each of which produces unique proteins in response to specific physiologic inputs, i.e., apoptosis. Apoptosis is a highly regulated form of programmed cell death that eliminates damaged or unwanted cells. However, if the apoptotic pathway fails, the consequences might be catastrophic. It may activate and deactivate various cellular signals to destroy cell functions [9]. Mitochondrial outer membrane permeabilization (MOMP), controlled by both pro- and antiapoptotic proteins, is associated with these different apoptotic situations [10]. It has been shown that several proteins in the cell have both pro and antiapoptotic activities and play a vital role in the regulation of apoptosis, such as B-cell lymphoma 2 (Bcl-2). These oversee cell stability via protecting mitochondrial membrane coherence to regulate complex interactions between its pro-apoptotic proteins, e.g., Bax (Bcl-2-associated X protein), Bak (Bcl-2 homologous antagonist killer), and antiapoptotic proteins, e.g., Bcl-xL (B-cell lymphoma extra-large) [11]. Thereby, the survival or death of cells is determined by the delicate balance between these proteins [12].

On the other hand, caspases are an essential family of cysteine proteases that play an important role in the process of apoptosis. Executioner caspases are activated in the terminal stage of the apoptotic pathway [13], and they are responsible for the actual execution of apoptosis [13]. Since antiapoptotic proteins like Bcl-2 are absent, cytochrome c can escape from the mitochondria and enter the cytoplasm, where it attaches to Apaf-1 and initiates the process that develops the apoptosome. This, in turn, causes the activation of Caspase-9, activating Caspase-3 and Caspase-7 in the following chain of events [14]. As a result, in some cases, the survival rate of chondrocytes may be reduced due to changes that de-regulate the apoptotic process [15]. The Bcl-2 also seems to suppress apoptosis by regulating and stimulating many promoters involved in the process of its downstream [16]. Following that, researchers revealed the maintenance of mitochondrial integrity during intrinsic apoptosis as a mediator of intense cytoprotective activities [17]. Moreover, Bax and Bak (members of the pro-apoptotic family) are essential for destabilizing the outer mitochondrial membrane and the eventual transfer of apoptogenic proteins that result in the activation of caspases due to the suppression of Bcl-2 protein [18]. Thereby, it causes the release of proteins from the gap between the inner and outer mitochondrial membranes, either directly or indirectly, that leads to the release of cytochrome c (Cyto C) and other soluble proteins into the cytoplasm [19].

Recent studies suggest that inflammasome activation is interlinked with apoptosis in specific contexts. For instance, the release of mitochondrial DNA, a hallmark of apoptosis, can activate a significant component of the inflammasome complex [20]. Furthermore, apoptotic cells like macrophages can inhibit inflammasome activation by clearing away phagocytic cells [21]. Overall, the interplay between apoptosis and inflammasome activation is a complex and dynamic process critical in regulating immune responses and maintaining tissue homeostasis. There are different disease conditions where uncontrolled apoptosis turned into a disaster, e.g., tibial dyschondroplasia (TD) [22]. Over the last decade, a tremendous breakthrough has been achieved in the perception of the cellular and molecular processes that increase the frequency of chondrocytes apoptosis and its underlying mechanisms [23, 24]. According to our previous research findings, chondrocytes apoptosis is dysregulated due to alterations in cellular signaling. It may involve deactivating antiapoptotic proteins such as beta cell lymphoma-2 over an extended period [15]. Maintaining the integrity of the mitochondrial membrane plays a crucial function in controlling cell survival and death. As a result, Bcl-2 dysregulation has been linked to the development of various disorders, including cancer, autoimmune and cardiovascular diseases [9]. Moreover, it has been verified in our previous research that the cleavage and activation of executioner caspases are responsible for considerable intracellular proteolysis, disruption of cellular functioning, and death of chondrocytes. The principal executioners are commonly thought to have functionally comparable functions within the cell death process due to their almost identical activity with Bcl-2 [25]. As a result, the depletion promotes death in chondrocytes through complementary pathways [26]. So, specific treatment for such disorders is essential to control these irregularities.

The neutralization of antiapoptotic subclasses of the Bcl-2 family and caspases is vital for regulating apoptosis in chondrocyte disorders. According to previous literature, the small molecule inhibitors regarding BH3-only proteins, act as an upregulator of Bax and Bak and are essential for inducing apoptosis. Multiple transcriptional and post-translational mechanisms tightly control their activity. These (BH3-only) proteins selectively bind to the hydrophobic receptor of antiapoptotic Bcl-2 family members, leading to Bax/Bak activation through direct or indirect activation models [27, 28]. Apoptosis is additionally goverened by the fact that Bcl-2 proteins can only bind to BH-3-only proteins to a limited extent [29]. Antisense oligonucleotide also targets by binding on the open reading frame of the Bcl-2 gene, causing mRNA degradation and hence resulting in the blocking of its expression at the protein level [30]. Moroever, peptides may potentially have implications for chondrocyte disorders associated with apoptosis. The peptide sequences that mimic BH-3-only proteins bind Bcl-2 with higher affinity than pro-apoptotic proteins and cause a reduction in their interaction. Free pro-apoptotic proteins can then stimulate the apoptotic cascade [31].

Chlorogenic acid (CGA) is a polyphenolic compound derived from natural plants such as honeysuckle, strawberry, coffee beans, and sunflowers [32]. It has a variety of pharmacological functions in antioxidant, anticancer, antibacterial, and anti-inflammatory activities [33]. It has been used as a dietary supplement to prevent tumorigenicity in several cancer research studies. Furthermore, in addition to its capacity to affect the growth and death of cancer cells, CGA has been proposed as a possible chemoprotective medication. It is due to its potential to postpone or counteract the incidence of chemical carcinogenesis [34]. According to the findings of research conducted by Zeng et al., CGA dramatically reduce tumor development, increase survival rate, and block pulmonary metastasis, all contributing to improve antitumor immunity [35]. Aside from these bioactivities, CGA can suppress cancer invasion and metastasis. It stop the transcription of MMP-2 and MMP-9 in HepG2 xenograft tissue of hepatoma [36]. Moreover, our recent study found that CGA enhances the expression of beta cell lymphoma-2 in thiram-affected chondrocytes by exerting a soothing role against the chondrocyte’s apoptosis [15]. The prominent role of CGA in protecting chondrocytes urged us to investigate its binding motif in facilitating the interaction with complimentary proteins in chondrocytes and the counter effect in case of complications regarding siRNA-based therapeutics. Hence, the study objectives were to investigate the mechanical involvement of the beta cell lymphoma-2 in upholding apoptosis and inflammasome, regulatory role in chondrocyte functioning, and their co-delivery role with Chlorogenic acid in vivo and in vitro models, and also to find out the possible therapeutic interventions of CGA in cartilage or chondrocyte related disorders.

## Material and method

### Experimental material

A random number of male and female day-old AA broilers (*n* = 140) were purchased from Henan Zhongzhou Co., Ltd. Chlorogenic acid (B20782) was obtained from Yuanye Bio Shanghai, China. Trizol® reagent (Invitrogen) was purchased from Thermo Fisher Scientific, USA. Quantitative PCR diagnostic kits based on reverse transcription were purchased from TransGen Biotech Co., Ltd. (Beijing, China). The miR-460a inhibitors, miR-460a mimics, Bcl-2 siRNA (si-Bcl2), and corresponding negative controls (NC) were designed and synthesized by GenePharma Co. (Shanghai, China). DAPI stain (4′,6-diamidino-2-phenylindole; 1 mg/mL; C0060) was obtained from Solorbio Life Sciences (Beijing, China). Hyperglycemic DMEM was purchased from HyClone, USA. Opti-MEM® I Reduced Serum Medium, Australian FBS, and Penicillin-Streptomycin (10,000 U/mL) were purchased from Gibco, Thermo Fisher Scientific, USA. The Collagenase (BS164-100 mg) and Hyaluronidase (BS171-100 mg) were purchased from Biosharp, Life Sciences, China.

### Construction of healthy and diseased models for the analysis of chondrocyte damage under beta cell lymphoma 2 and miR-460a expression

**Fig. 1:**
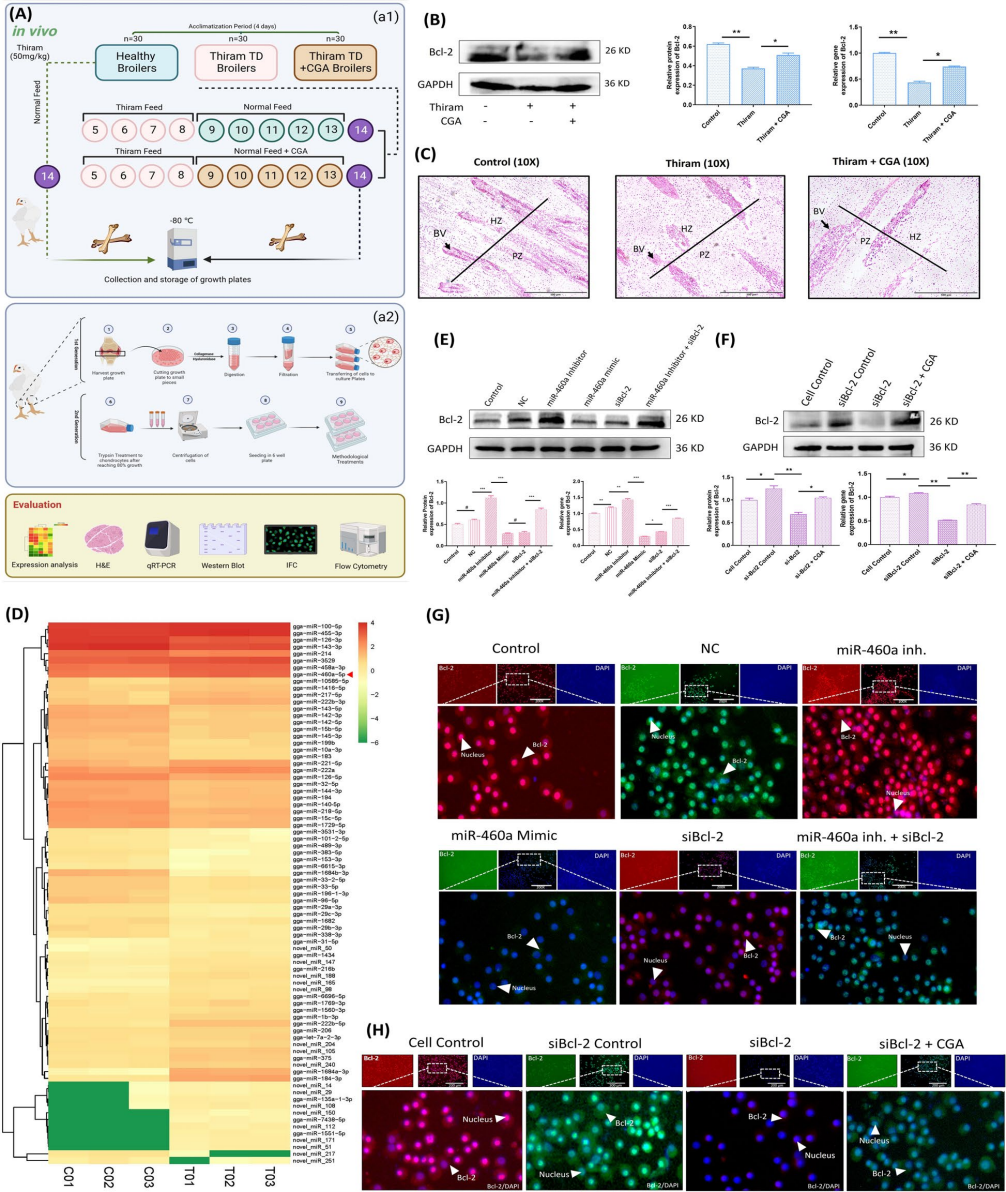
The expression analysis of B-cell lymphoma 2 in in vivo and in vitro models of chondrocytes. **A** The layout of in vivo and in vitro models for analyzing chondrocyte damage. **B** The western blot analysis comparing B-cell lymphoma 2 (Bcl-2) expression levels in a thiram-induced in vivo model. **C** Histological evaluation of growth plate sections stained with H&E reveals distinct vascular patterns and chondrocyte morphology in different experimental groups. (BV: Blood vessel, PZ: Proliferative Zone, HP: Hypertrophic zone). **D** Expression verification of miR-460a in tibial growth plate through high-throughput sequencing. **E**,** G** The transfection interpretation of B-cell lymphoma 2 in various groups using a Western blot analysis, immunofluorescence assay, and RT-qPCR. The transfection shows a significant difference, indicating a potential role of Bcl-2 in chondrocyte progression. **F**,** H** Quantitative analysis of the co-delivery effect of siBcl-2 and CGA. Notably, co-delivery with CGA demonstrated an enhanced expression of Bcl-2, suggesting a potential role of CGA in modulating Bcl-2 expression (Magnification, 200X).“^*, #^” shows the significance level among groups. ^*^p < 0.05, ^**^ *p* < 0.01, ^***^ *p* < 0.001, ^#^ *p* > 0.05

The healthy AA broilers were randomized to control, thiram, and CGA groups of 10 birds each (consisting of 10 birds with three replicates, totaling 30 birds). The control groups received a standardized diet for the entire experiment following a four-day acclimatization period. In the thiram group, the birds were fed a diet containing 50 mg/kg of thiram for the next 4 days, after which they were switched back to the standard diet. For the CGA group, the birds were initially fed the same thiram dose for 4 days, and then CGA was administered through drinking water for the remaining time of the experiment. Throughout the investigation, all the chickens had unrestricted access to drinking water. On the 14th day, samples of tibial bones from all groups were collected and stored in a − 80 °C refrigerator for later experiments, i.e., High-throughput sequencing, H&E Staining, Western blotting, and RT-qPCR (Fig. 1A, a1).

### Chondrocytes culturing and transfection for downstream signaling pathways

The growth plates were cut into 1 mm^3^ pieces, referred to digestion with digestive media in a humidified shaker with 5 % CO_2_ at 37 °C for 8–12 hours. After that, the digested media was filtered through a sieve with a 70 μm size to collect chondrocytes. Finally, cells were put into a culture flask (T25) at 2 × 10^5^ cells per flask of culture medium (maximum 5 mL total volume) after a proper centrifugation and washing process [4]. The culture media was prepared with 89% hyperglycemic DMEM, 10% FBS (fetal bovine serum), and 1% penicillin-streptomycin. The digestive media was prepared as the standard culture media by adding digestive enzymes, i.e., collagenase and hyaluronidase. To subculture the cells, 0.25% trypsin-EDTA (Gibco, USA) was used to digest cells for 2 min. Then, cells were sub-cultured to the new culture plates according to the further experimental necessities (Fig. 1A, a2). When the cells achieved a second generation with a 70–75% density, we transfected with miR-460a mimics, inhibitors (2.5 μl of 2 μM), siBcl-2 (0.5-4 μg/μL of 20 μM), and their normal controls by using Lipofectamine 2000TM for 24 hours. For the treatment aspect, we employed CGA (Chlorogenic acid) as a pre-treatment at a dose rate of 40 μg/mL, based on the dosage utilized in our previously published study [26]. After applying different treatments, the culture media with Dulbecco’s Modified Eagle Medium (DMEM) was supplemented with 10% fetal bovine serum (FBS). This medium was maintained for 72 hours to allow for the collection of samples.

### Antibodies

The following antibodies were used: anti-Bcl-2 pAb (Bioss, Proteintech Group), anti-Cytochrome C Rabbit pAb (ABclonal Technology), anti-Bax Rabbit pAb (Wanlei Biotechnology Co., Ltd), anti-Bak Rabbit pAb (ABclonal Technology), anti-caspase-3 Rabbit pAb (ABclonal Technology), anti-caspase7/cleaved-caspase7 Rabbit pAb (ABclonal Technology), IL-1β (Wanleibio Biotechnology Co., Ltd), anti-aggrecan Rabbit pAb (ABclonal Technology), Coll1A2 Rabbit pAb (ABclonal Technology), anti-GAPDH Rabbit pAb (ABclonal Technology), HRP goat anti-rabbit IgG (ABclonal Technology), Alexa Fluor® 488 labeled goat anti-rabbit IgG (Servicebio Technology Co., Ltd), Cy3 labeled goat anti-rabbit IgG (Servicebio Technology Co., Ltd).

### Experimental layout (in vitro)

In the first experiment, we used a miR-460a inhibitor, a miR-460a mimic, siBcl-2, and a combination of the miR-inhibitor and siBcl-2 to modulate Bcl-2 expression in chondrocytes. The normal and control groups served as baselines against which physiological parameters were measured. The main objective of this investigation was to look into the impact of Bcl-2 expression on apoptosis and inflammasome activation in chondrocytes. In the second experiment, we used small interfering RNA (siRNA) to target Bcl-2 and selectively assess its co-delivery impact with CGA. We examined the impact of CGA on Bcl-2 expression levels in chondrocytes in an effort to gain more insight into the molecular mechanisms underlying the interaction between CGA and Bcl-2. Firstly, we compare the results of the treatments by employing the miR-460a inhibitor and mimic (a microRNA known to regulate Bcl-2 expression), confirmed by our previous study [37].

Furthermore, the expression of Bcl-2 in chondrocytes was targeted and precisely suppressed using siBcl-2. We used a variety of quantitative and qualitative tests to assess the impacts. Apoptosis and inflammasome activation in chondrocytes were assessed using recognized methods such as flow cytometry, immunofluorescence assays, and Western blot analyses. These experiments supplied consistentt data to analyze the effect of Bcl-2 expression modulation on critical cellular functions. Next, we looked at the impact of CGA and siBcl-2 co-delivery in chondrocytes to investigate the effect of CGA on Bcl-2 expression levels. CGA, a potentially medicinal compound, has been shown to interact with Bcl-2 previously. The study used techniques including real-time PCR and protein analysis to evaluate changes in Bcl-2 expression after CGA therapy. We carefully controlled variables that may confuse the results throughout the studies, including adequate positive and negative controls, multiple experiments to guarantee repeatability, and statistical analysis to establish the significance of the reported effects (Fig. S1).

### Detection of cell apoptosis through flow cytometry

After transfection, the cells were trypsinized with EDTA-free trypsin for collection in 1.5 mL centrifuge tubes. Then, cells were stained using Annexin V-FITC and Propidium Iodide (PI) staining solutions for 15 minutes at room temperature according to the manufacturer’s instructions (Cat No. BA1250, Nanjing Bibo Biological Co., Ltd.), followed by staining using 1 mL of HEPES buffer. After all procedural formalities, apoptosis was detected at 485 nm using FITC-A fluorescence (525 nm) and PI fluorescence (620 nm) with Novacyte Flow Cytometer (Acea Bioscience, USA).

### Immunofluorescence

Chondrocytes from different groups were washed thrice with PBS before being treated with 4% paraformaldehyde for 15 minutes. Then, Triton X-100 (0.5%) for 20 minutes, H_2_O_2_ (0.3%) for 15 minutes, and the primary antibodies for 12 hours were applied respectively to the cells. Later on, the secondary immunofluorescent antibodies were used for 30 minutes, followed by five washes with phosphate-buffered saline + 0.05% TWEEN® 20. After that, the cells were incubated with DAPI for 5 min. Finally, a fluorescent microscope was used to observe the chondrocytes after being washed.

### RNA extraction and RT-qPCR

Total RNA was isolated from the chondrocytes using Trizol (Life Technologies, USA) as directed by the manufacturer. Thermo Fisher Scientific’s Nanodrop 2000 analyzer (at 260 nm and 280 nm) and formaldehyde gel electrophoresis were used to validate the integrity and quantity of RNA [26]. All samples had absorption ratios (260/280 nm) between 2.0 to 2.3. After that, random primers were used to reverse transcribe 1 μg of total RNA using M-MLV reverse transcriptase (Accurate Biotechnology) [38]. Following a two-minute incubation at 42 °C, a polymerase chain reaction (PCR) was performed in 20 mL of RT reaction products (37 degrees Celsius for 15 minutes; 85 degrees Celsius for 5 seconds). Then, qRT-PCR was performed at the following temperatures using SYBR Green Plus Reagent Kit (Accurate Biotechnology): 1 cycle at 95 degrees Celsius for 30 seconds, 35 cycles at 95 degrees Celsius for 5 seconds, and 30 seconds at 60 degrees Celsius. Finally, target gene mRNA levels were evaluated employing a Light Cycler 96 instrument (Roche, Switzerland) using specific primers with their controls (GAPDH or U6) (Tables S1 and S2). The 2 − ΔΔCT comparison approach and GAPDH threshold cycle (CT) values were used to examine the relative expression of each target gene [39]. Our previous study contains a list of the primers utilized in this study [15].

### Western blotting

A radioimmunoprecipitation assay buffer (RIPA buffer) pre-treatment with protease inhibitors was used to lyse chondrocytes from all groups to assess the effects of miR-460a and siBcl-2 on associated cell signaling proteins. Centrifugation was utilized to remove cell debris from the samples. The total protein contents of chondrocytes (nuclear and cytoplasmic) were determined using a Bicinchoninic Acid (BCA) Protein Assay Kit (Thermo Fisher Scientific, USA) with a BSA (Bovine Serum Albumin) as a protein standard. A 30 μL (containing about 50 μg protein per sample) of each sample per well was used [40] in 6, 12, and 15% of SDS-PAGE. After that, the proteins were blotted onto polyvinylidene fluoride membranes and then blocked with skim milk at a concentration of 5% for 2 hours at room temperature. At four degrees celsius, the membranes were exposed to the primary antibodies for 12 hours (overnight). Following this step, the membranes were washed with TBST and then incubated with the secondary antibody for an hour. Following five washes with TBST, each band’s density was determined using an enhanced chemiluminescence (ECL) substrate (Item No. BL520A; Biosharp, Life Sciences, China). For Preparing the ECL Working Solution, equal volumes of ECL Substrate A and B were mixed. Then, an appropriate volume was dropped on the membranes. The exposure time was adjusted according to the luminous intensity of each membrane. The resulting TIFF images were imported into Fusion software (VIBER Biotech, France) for analysis. For this purpose, each band was selected by drawing a rectangular box around a gray area to measure its mean value. The data were expressed as the mean relative value of the protein of interest and its loading control.

### Statistical analyses

The statistical analysis was carried out using the SPSS 22.0 program (SPSS Inc., Chicago, USA), with a one-way analysis of variance (ANOVA). Post hoc tests, including the Tukey and Least Significant Difference (LSD), were used to identify group differences. Both tests thoroughly analyzed the data to verify their validity and correctness. Throughout the investigation, normality, homogeneity of variances, and suitable sample sizes, were evaluated. Moreover, the results were given with measurements of both central tendency (means) and dispersion (standard deviations or standard errors) to provide the data with a complete representation. We could use these statistical techniques to identify significant differences between treatment groups and make inferences regarding the results of varying Bcl-2 expression levels in chondrocytes.

## Results

### Expression analysis of B-cell lymphoma 2 with CGA in the chondrocytes of in vivo and in vitro models

In this study, we set out to see if CGA might be used as a treatment for chondrocyte damage. To further understand the processes, involving the impact of CGA on Bcl-2 expression, we employed CGA in both in vivo and in vitro models. As a first step in our study, we looked at how Bcl-2 was expressed in an animal model. For this purpose, thiram was used, which showed a significant drop in Bcl-2 expression. From a treatment perspective, a group was given thiram along with CGA to see how it changed the expression of Bcl-2. Surprisingly, the CGA group had more expression (Fig. 1B).

Moreover, we looked at histology micrographs to learn more about the changes. According to the findings, the thiram group lacks blood vessels within the growth plate, indicating limited infiltration across growth plate zones. Contrarily, CGA therapy resulted in an increased dispersion of blood vascularization, with enhanced infiltration within the growth plate. This vascular dysfunction showed the advesre effects of thiram, which might have serious consequences for the chondrocytes. Moroever, the appearance of the chondrocytes in the thiram group was distorted, disorganized, and fragmented. The extent of damage to chondrocytes was highlighted by the fact that many dead cells did not have nuclei or had deformed and necrotized nuclei (Fig. 1C).

We modulated the expression of Bcl-2 in the growth plate via miR-460a, based on our earlier results that demonstrated a relationship between Bcl-2 and miR-460a. The expression patterns of miR-460a in the normal and thiram-induced groups were compared using high-throughput sequencing. Significant variations in miR-460a expression were found in our research, suggesting that miR-460a regulates Bcl-2 expression and chondrocyte functions (Fig. 1D). The results gave a critical viewpoint about the molecular pathways responsible for chondrocyte damage and the probable importance of siRNA-based therapeutics. The in vitro studies were conducted to grasp the complicated processes better and to explore the dynamic between Bcl-2, miR-460a, and restorative therapies. Specifically, we investigated the effect that miR-460a and siBcl-2 on Bcl-2 by using a co-transfection approach. Our objective was to ascertain the effects separately and in combination. Through a variety of comprehensive investigations, including western blotting, RT-qPCR, and immunofluorescence assays, we investigated the Bcl-2 protein expression among the different experimental groups. The findings demonstrated a substantial drop in the groups that were siBcl-2-transfected with miR-460a mimic and siBcl-2 alone. This pronounced decrease in Bcl-2 expression provided strong evidence for the regulatory role of miR-460a in modulating Bcl-2 levels and influencing chondrocytes survival and functions (Fig. 1E-G).

For keeping all these aspects, we further validated the influence of CGA on Bcl-2 expression; we conducted additional experiments involving siBcl-2 transfection with CGA pre-treatment. Surprisingly, our results revealed a notable decrease in the inhibitory effect of siBcl-2 when chondrocytes were pre-treated with CGA (Fig. 1F-H). This unexpected finding suggested that CGA may exert its influence through the regulation of miR-460a, thereby mitigating the inhibitory effects of siBcl-2. These results were consistent with our previously published study, which highlighted the potential role of CGA in modulating Bcl-2 expression and promoting chondrocytes survival [37].

### B-cell lymphoma 2 exerts a protective role in chondrocyte’s apoptosis with down-regulation of Bax and Bak mediated flux of cytochrome C under CGA

**Fig. 2:**
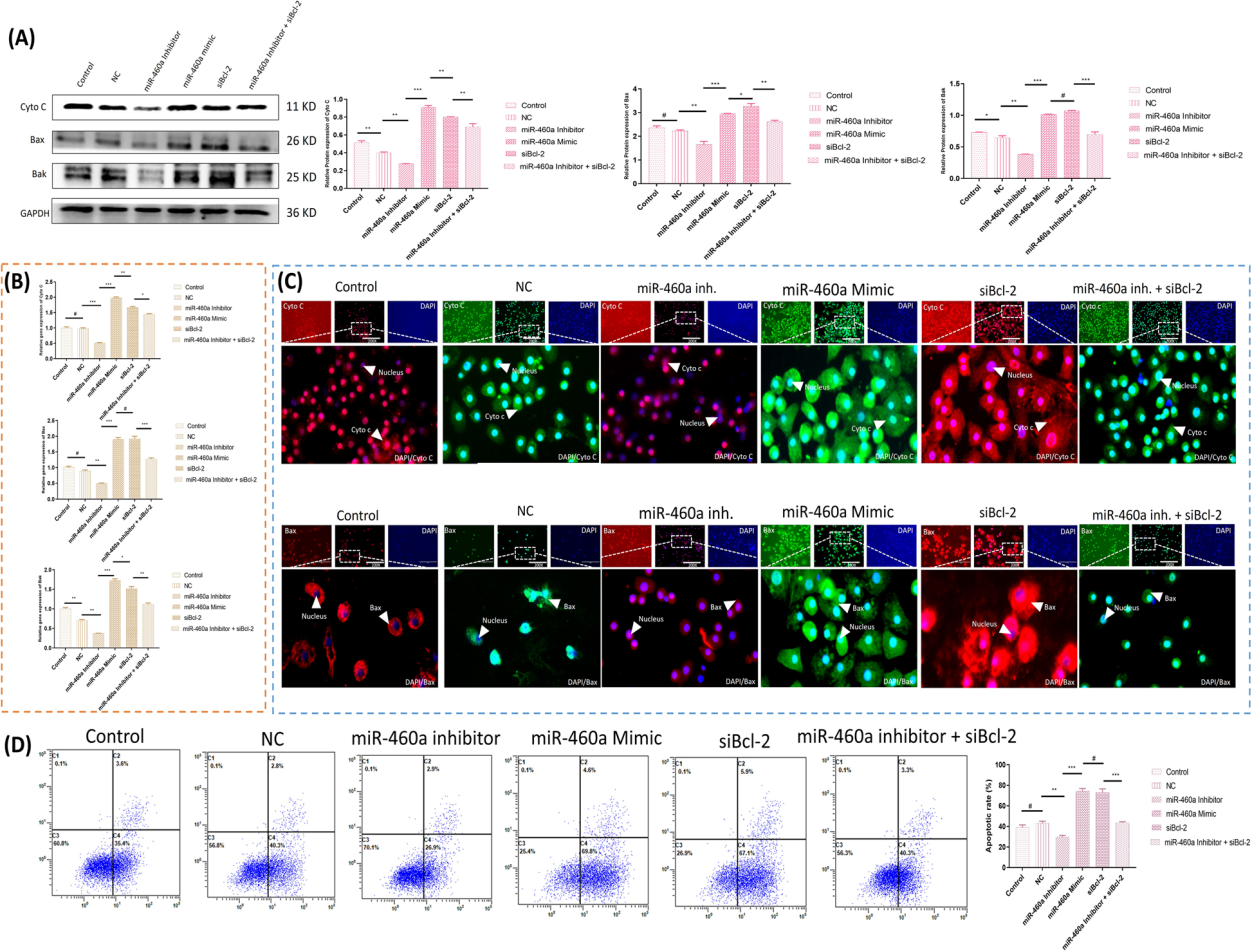
The expression profile of Cytochrome C (Cyto C), Bax, and Bak under the repercussions of miR-460a and siBcl-2. **A** The mechanism of Bax and Bak permeabilization of mitochondria during apoptosis, causing Cyto C to release in the cytoplasm. **B**,** C** Different groups’ interpretation of transfected chondrocytes was confirmed by Western blot assay and RT-qPCR in different groups. Bar graphs show the proteins and gene expression levels in various groups with varying transfection conditions. **D** Immunofluorescence assay reveals a significant regulation of Cyto C, Bax, and Bak, visualized through red and green fluorescence, while nuclear staining was depicted by blue fluorescence. **E** Flow cytometry analysis of chondrocytes of different groups. The miR-460a-inhibitor showed a low level of apoptotic cells, while the miR-460a-mimic group exhibited a higher level of apoptotic cells. The different sections represent distinct populations of apoptotic cells with varying intensities. The results were expressed in means ± SD, repeated three times for each group (*n* = 3). “^*, #^” shows the significance level among groups. *p < 0.05, ^**^ *p* < 0.01, ^***^ *p* < 0.001, ^#^ *p* > 0.05

To provide detailed insights into the molecular mechanisms underlying the provocative role of Bcl-2 mediated siBcl-2 in chondrocyte apoptosis, explicitly involving the upregulation of Bax and Bak, with the flux of Cytochrome C (Cyto C), we conducted co-transfection experiments using siBcl-2 and miR-460a. Our results demonstrated that the suppression of Bcl-2 resulted in enhanced levels of Cyto C (Fig. 2A). Additionally, we explored the relationship between miR-460a and the targets, as mentioned earlier, by co-transfection. The expression level of Cyto C was significantly influenced by miR-460a. Similarly, the immunofluorescence assays also revealed an increased Cyto C levels in cells transfected with miR-460a mimic and siBcl-2 (Fig. 2B-D). Moreover, we observed a substantial decrease in apoptosis when using a miR-460a inhibitor compared to the negative control group (*p* < 0.001). Notably, the apoptotic rate of chondrocytes co-transfected with siBcl-2 and miR-460a inhibitor significantly decreased compared to the siBcl-2 group (*p* < 0.001), suggesting the role of Bcl-2 in reducing apoptosis in chondrocytes (Fig. 2E).

**Fig. 3:**
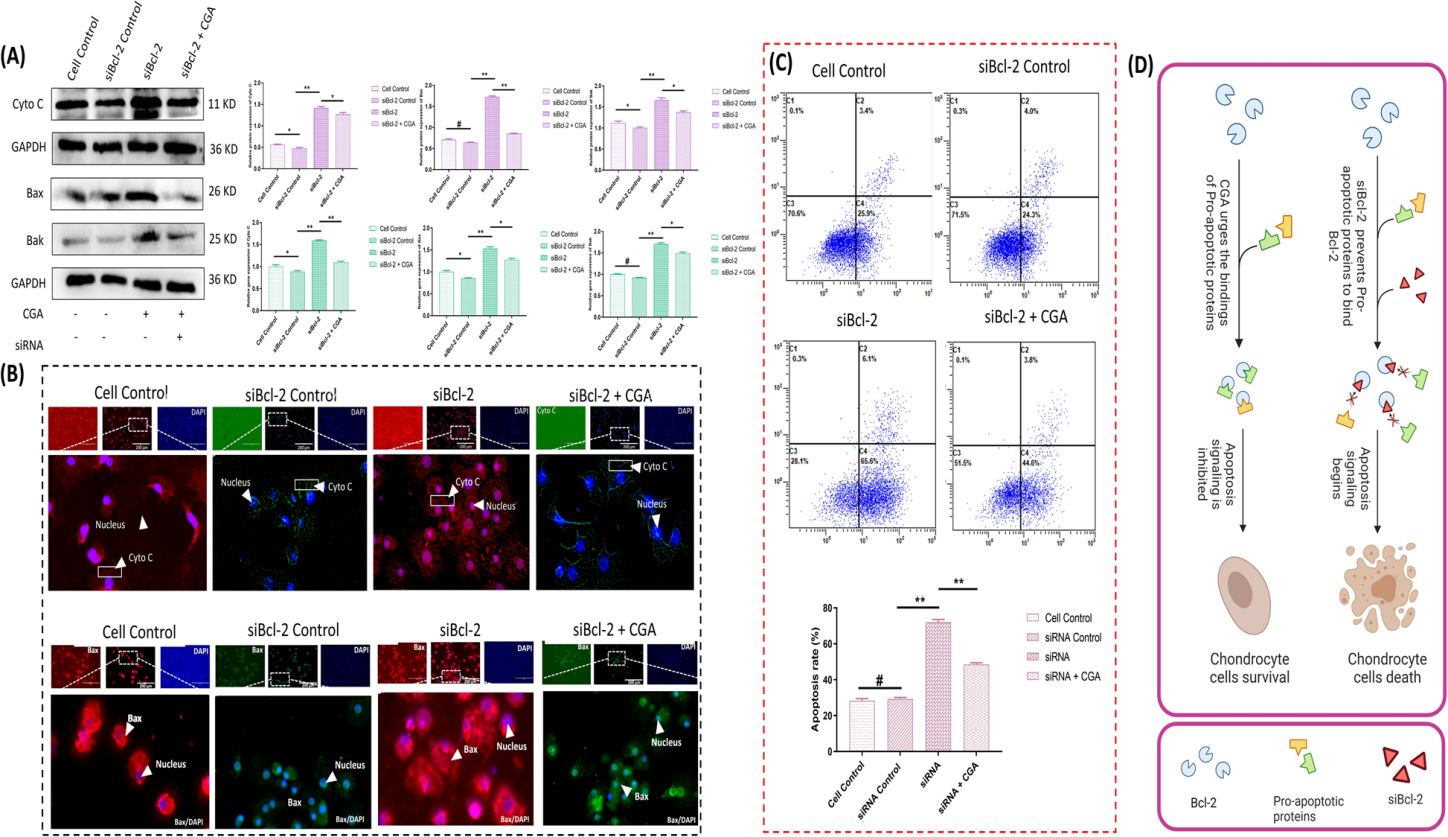
The expression profile of Cytochrome C (Cyto C), Bax, and Bak among different groups under the co-delivery effect of siBcl-2 and CGA. **A** Comparative analysis of chondrocyte expression levels in various groups through Western blot and RT-qPCR techniques. **B** Immunofluorescence assay demonstrating the expression levels in chondrocytes. The figure shows representative images capturing the immunofluorescent signals, indicating the localization and abundance of the target proteins in each group (Magnification, 200X). **C** Analysis of apoptosis through flow cytometry and their statistical comparison through one-way ANOVA with SPSS 20.0 software (SPSS Inc., Chicago, IL). **D** The prediction of CGA role in chondrocytes survival via upholding Bax/Bak mitochondrial permeabilization. The results were expressed in means ± SD, repeated three times for each group (*n* = 3). “^*, #^” shows the significance level among groups. *p < 0.05, ^**^ *p* < 0.01, ^***^ *p* < 0.001, ^#^ *p* > 0.05

Further, we used siBcl-2 in combination with CGA pre-treatment to investigate other therapeutic avenues.. Statistical analysis revealed a relatively higher expression of Bcl-2 in the CGA pre-treatment group, confirming the potential of CGA in enhancing Bcl-2 expression (Fig. 3A, B). Additionally, the apoptosis level showed a lower percentage of apoptotic cells in the CGA group compared to the siBcl-2 group. Flow cytometry analysis based on Annexin-V/PI staining indicated a significantly higher rate of cell death in chondrocytes treated with siBcl-2 alone (*p* > 0.05). However, the apoptosis rate was significantly reduced when siBcl-2 was combined with CGA treatment (*p* > 0.05), indicating the protective impact of CGA against siBcl-2-induced apoptosis (Fig. 2C). According to our hypothesis, the release of Cytochrome C (Cyto C) is facilitated by short interfering RNAs against Bcl-2 due to pro-apoptotic proteins being translocated to the mitochondria. Also, here we demonstrated that Cyto C is released from mitochondria in feedback to siBcl-2-mediated chondrocytes. We employed CGA as a pre-treatment to ensure the involvement of Cyto C in chondrocytes and investigated the proteins and genes associated with it. As a result, treatment with 40 μg/mL CGA conspicuously decreased the level of apoptotic cells caused by the depletion of Bcl-2. The immunofluorescence assay also revealed an increased fluorescence of Cyto C in siBcl-2-transfected cells, indicating chondrocyte apoptosis through mitochondrial-dependent signaling under pro-apoptotic-mediated mitochondrial membrane degradation and the release of Cyto C. Hence, these findings confirm that the Bcl-2 subsequently reduces chondrocytes death through a mitochondrial-dependent pathway, which involves the Bax/Bak-mediated flux of Cyto C. Moreover, the treatment, e.g., CGA, might be an option for Bcl-2, involving conditions (Fig. 3D).

### Effect of B-cell lymphoma 2 (Bcl-2) expression on chondrocyte’s morphology and its related markers

**Fig. 4:**
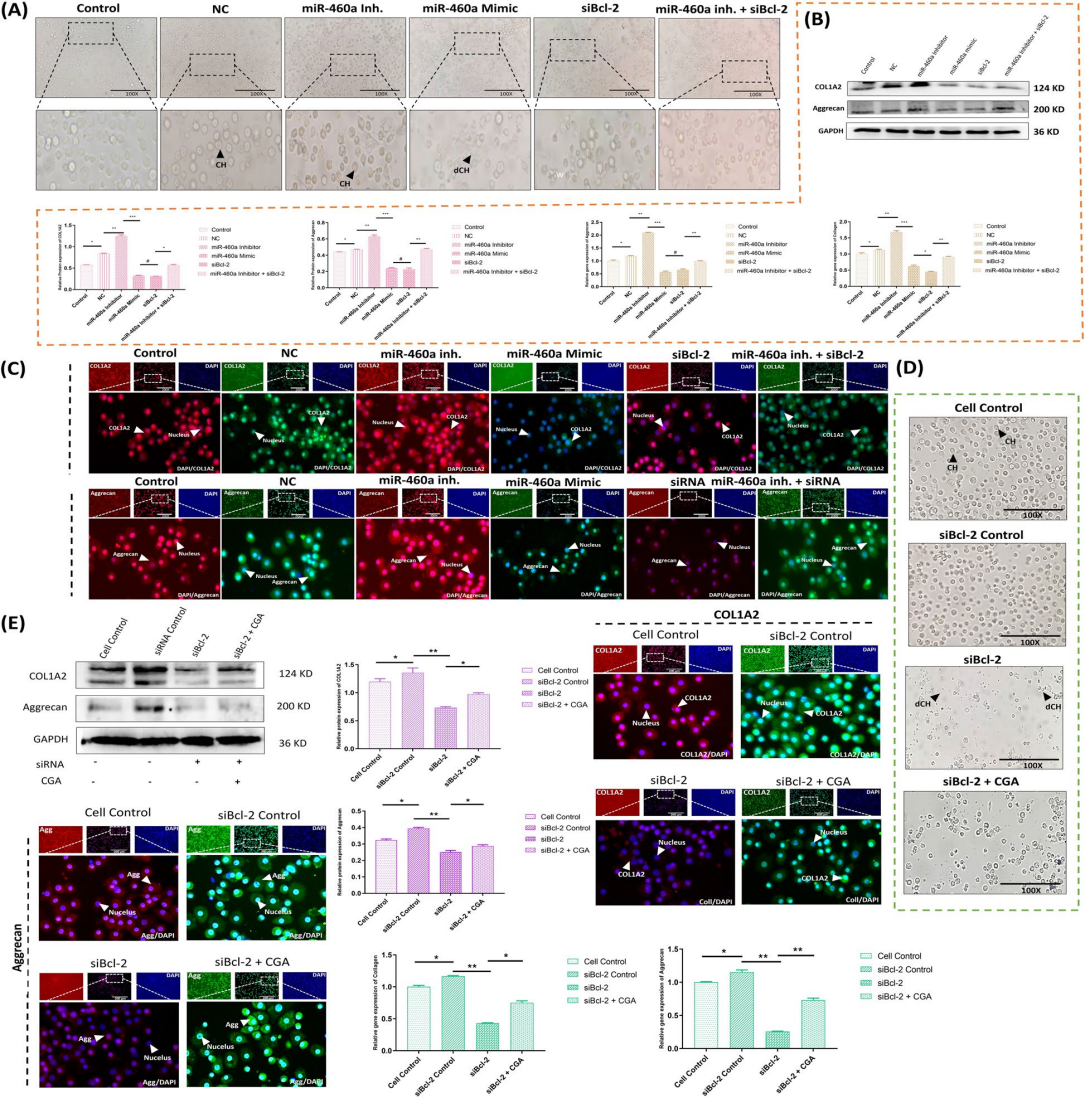
The morphological analysis of chondrocytes and their related markers (COL1A2, Aggrecan) under the repercussions of miR-460a and CGA. **A** The outward appearance of chondrocytes as seen via an inverted microscope (100X) in co-transfected groups. **B**,** C** The expression of target proteins and genes (COL1A2 and aggrecan) through Western blot assay and RT-qPCR, and immunofluorescence assay (200X) in co-transfection, showing a significantly decreased expression of COL1A2 and Aggrecan expression in miR-460a mimic and siBcl-2 groups. The miR-460a mimic and siBcl-2 transfected cells exhibited low expression levels. In contrast, the miR-460a inhibitor and miR-460a inhibitor + siBcl-2 groups showed a significantly higher expression level. **D** Morphological analysis of chondrocytes in the co-delivery effect of siBcl-2 and CGA under an inverted microscope (Magnification, 100X). **E** The expression of COL1A2 and Aggrecan in chondrocytes by western blot, RT-qPCR, and immunofluorescence assay (Magnification, 200X) in Cell Control, siBcl-2 Control, siBcl-2, and siBcl-2 + CGA. Bar graphs show the collation of proteins and gene expression with statistical analysis. The results were expressed in means ± SD, repeated three times for each group (*n* = 3). ^*^p < 0.05, ^**^ *p* < 0.01. “#” shows the non-significance between groups. (CH; normal chondrocyte, dCH; damaged chondrocyte)

After a 12-hour incubation at 37 °C, the microscopic analysis of chondrocytes revealed distinct differences among different groups. In both the Control and miR-460a inhibitor groups, chondrocytes appeared round, prominent, and intact. However, in the miR-460a mimic and siBcl-2 groups, the chondrocytes seemed shrunken, smaller, and damaged, exhibiting a different cell shape and dispersed arrangement. In addition, the chondrocytes that were co-transfected with a miR-460a inhibitor and siBcl-2 had a uniform look, were plump, had a paving stone-like shape, and were arranged in a tightly organized pattern. It is important to note that there was no significant association between the control group and the group treated with miR-460a mimic and siBcl-2 (Fig. 4A). We studied the expression of marker proteins and genes linked with chondrocytes, namely COL1A2 (collagen type I alpha 2) and ACAN (aggrecan encoding gene), so we could better understand the impact. We used different types of analysis, including western blotting, real-time quantitative PCR, and immunofluorescence, to see whether there were any changes in the expression levels. The results demonstrated that compared to the control group, which did not receive either the miR-460a mimic or the siBcl-2, the treated groups dramatically lowered the expression of both COL1A2 and ACAN. However, when chondrocytes were co-transfected with a miR-460a inhibitor and siBcl-2, the expression levels of COL1A2 and ACAN were within the normal range (Fig. 4B, 4C). These findings provide a more detailed understanding of the morphological changes observed in chondrocytes following different treatments and the corresponding alterations in the expression of crucial marker proteins and genes. In the second experiment related to the therapeutic efficacy of CGA, the chondrocytes were found to be spherical and conspicuous in both control groups but shrunken, tiny, and broken in the small interfering RNA (siBcl-2) group. Surprisingly, chondrocyte morphology in the CGA pre-treated group was almost normal, suggesting CGA impact against siBcl-2-transfected chondrocytes (Fig. 4D). Moreover, we noticed a lower expression of COL1A2 and ACAN in the siBcl-2 mediated group, while the expressions in the treatment group (siBcl-2 + CGA) were relatively higher. Another important observation was that COL2A1 and ACAN expression in the siBcl-2 group was barely detectable over immunofluorescence assay (Fig. 4E). Thus, these findings imply a potential relevance for chondrocyte differentiation and function with or without CGA.

### B-cell lymphoma 2 knockdown modulates Casp3/Casp7-mediated apoptosis

**Fig. 5:**
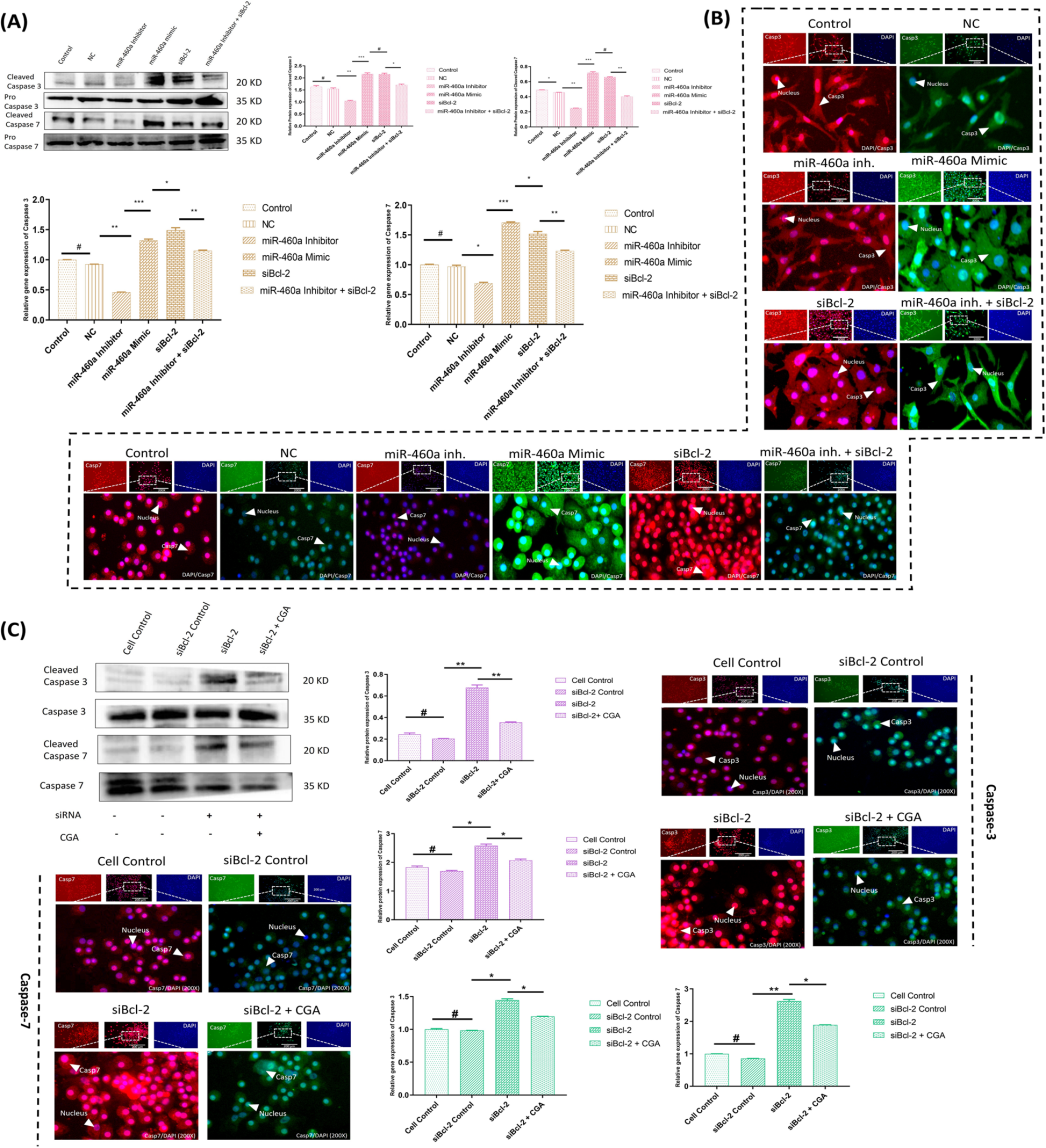
Role of executioner caspases (i.e., Casp3 and Casp7) under the repercussions of miR-460a and CGA. **A** The interpretation of Casp3 and Casp7 in chondrocyte apoptosis following transfection was elucidated through Western blot assay and RT-qPCR analyses across different experimental conditions. **B** Immunofluorescence assay of Casp3 and Casp7, labeled with specific antibodies (200X). The miR-460a mimic and siRNA-based Bcl-2 transfected cells exhibited higher protein expression levels, as shown by the higher fluorescence signal. In contrast, the miR-460a inhibitor and miR-460a inhibitor + siBcl-2 groups showed a significantly lower expression level. **C** The expression level of Casp3 and Casp7 in chondrocytes through western blot, RT-qPCR, and immunofluorescence assay (Magnification, 200X) in co-delivery of siBcl-2 and CGA. Bar graphs show the proteins and gene expression levels in various groups with varying transfection conditions. The error bars represent the standard deviation of the mean. “*” shows the level of significance among groups. ^*^p < 0.05, ^**^ *p* < 0.01. “#” shows the non-significance among groups

Executioner caspases are thought to be triggered during apoptosis [41]. With this understanding, we formulated a hypothesis regarding the involvement of Bcl-2 in the activation of executioner caspases. Consequently, we investigated the role of miR-460a in regulating executioner caspases, specifically Casp3/Casp7, concerning their influence under Bax/Bak-mediated apoptosis in mitochondria. To examine the accumulation of these mitochondrial proteins (Casp3 and Casp7), we divided the experimental groups into control, standard control, miR-460a inhibitor, and miR-460a inhibitor + siBcl-2 groups. Comparing the levels of Casp3 and Casp7 proteins in the miR-460a mimic and siBcl-2 groups, we observed a significant enhancement in their expression. Additionally, the gene expression levels of these proteins were found to be higher compared to the control groups. This substantial upregulation of Casp3 and Casp7 appeared to be driven by the Bax and Bak mediated influence of Cytochrome C, facilitating mitochondrial membrane perturbation in the group treated with the miR-460a mimic (Fig. 5A). We further employed an immunofluorescence assay to analyze the expression patterns. The miR-460a mimic and siBcl-2 groups exhibited significantly higher expression levels of Casp3 and Casp7 compared to the control group, miR-460a inhibitor group, and miR-460a inhibitor + siBcl-2 group (*p* < 0.05) (Fig. 5B). Based on these findings, we conclude that the augmented expression of Casp3 and Casp7, mediated by Bax/Bak mediated flux of Cyto C, correlates with the potential disruption of the mitochondrial membrane. Additionally, it was observed that treatment with CGA significantly attenuated the formation of these caspases. The lower levels of these caspases indicated that CGA treatment inhibited the degrading activity of chondrocytes during siBcl-2 transfection. In the CGA-mediated group, the immunofluorescence assay similarly indicated a substantial reduction in the expression of caspases. Intriguingly, the results of our research showed that CGA exerted a significant influence on the development and operation of executioner caspases. In particular, we found that the concentrations of Casp3 and Casp7 dropped significantly when CGA was present (Fig. 5C). Based on these findings, CGA has a potent inhibitory impact on destroying chondrocytes after siBcl-2 transfection. In addition, the results of our immunofluorescence study provided further evidence that CGA has an inhibitory effect on these caspases. When compared to the expression levels of Casp3 and Casp7 in the other experimental groups, such as the control group, the miR-460a inhibitor group, and the miR-460a inhibitor group with siBcl-2, we found that the CGA-mediated group had a much lower level of both proteins. In general, our results provide insight into the extraordinary potential of CGA as a therapeutic agent for modifying the processes of chondrocyte damage. By effectively reducing the formation and activity of executioner caspases, CGA demonstrates promising prospects for preserving chondrocyte integrity and function during siBcl-2-based interventions.

### Suppression of Bcl-2 regulates the inflammasome pathway, imploring upstream IL-1β production

**Fig. 6:**
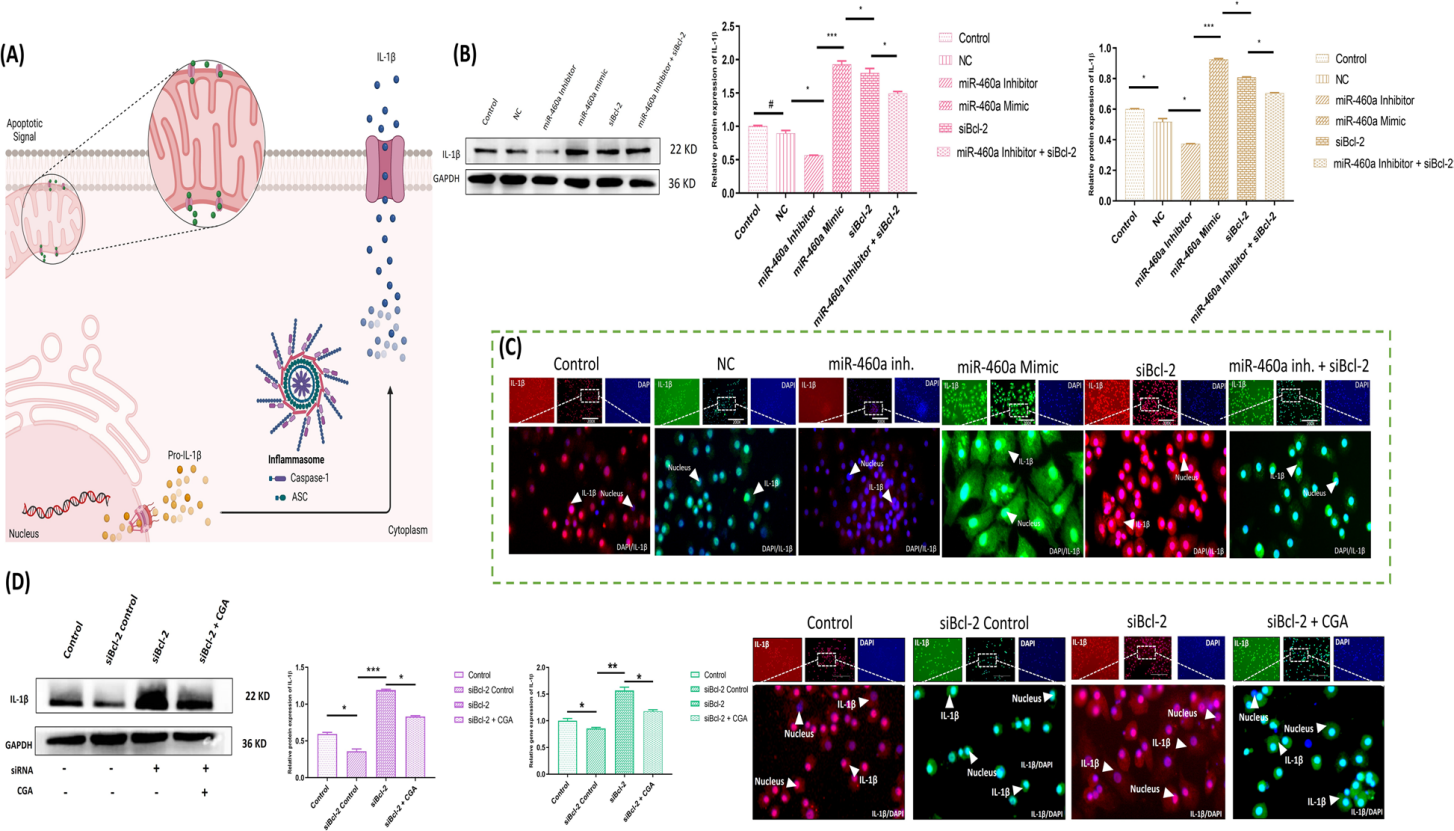
Investigation of imploring upstream of IL-1β production in chondrocytes. **A** The mechanism of IL-1β formation during the apoptotic signal. **B** The interpretation of IL-1β, confirmed by Western blot assay and RT-qPCR in different groups. **C** Immunofluorescence assay of IL-1β, labeled with a specific antibody (200X). The miR-460a mimic and siBcl-2 transfected cells exhibited higher protein expression levels, as shown by the higher fluorescence signal. The miR-460a inhibitor and miR-460a inhibitor + siBcl-2 groups, on the other hand, had considerably lower expression levels. **D** The figure indicates the findings of the Western blot, RT-qPCR, and immunofluorescence assays, which show the levels of IL-1β expression in chondrocytes under various treatment conditions (Control, siBcl-2, siBcl-2 + CGA). The siBcl-2 group had lower Bcl-2 protein expression, suggesting effective IL-1β upregulation. However, IL-1β expression decreased in the co-delivery group, demonstrating CGA’s potential for efficiently attenuating IL-1Β maturation. Bar graphs show the proteins and gene expression levels in various groups with varying transfection conditions. The error bars represent the standard deviation of the mean. “*” shows the level of significance among groups. ^*^p < 0.05, ^**^ *p* < 0.01. “#” shows the non-significance among groups

In the first part of the experiment, we aimed to investigate the impact of siBcl-2 and miR-460a on the expression levels of IL-1β at both mRNA and protein levels in chondrocytes. By revealing possible treatment methods for chondrocyte-related conditions, our results gave interesting insights into the relationship between miR-460a, Bcl-2, and IL-1β expression in chondrocytes. To begin, we found that IL-1β expression in chondrocytes was significantly increased with overexpression of miR-460a. The miR-460a’s probable participation in inflammatory processes inside chondrocytes is highlighted by this study, which implies that it plays a regulatory function in the induction of IL-1β. However, IL-1β maturation is linked to the upregulation of Bcl-2 due to its inhibition. Further evidence for its function in controlling IL-1β is provided by the observed reduction in miR-460a and Bcl-2 expression following inhibition, suggesting its potential as a therapeutic target for managing IL-1β-mediated inflammatory responses in chondrocytes. Surprisingly, we discovered that co-transfecting siBcl-2 with a miR-460a inhibitor decreased the production of IL-1β.This finding raises the possibility of relationship between Bcl-2 overexpression and elevated IL-1β maturation (Fig. 6A-C). We transfected siBcl-2 with pre-treated CGA’s chondrocyte to assess the therapeutic potential of a co-delivery method. Our data showed that the co-delivery of CGA (the intervention of interest) and siBcl-2 efficiently suppressed chondrocyte apoptosis, suggesting a useful therapeutic strategy for treating chondrocytes assocaited conditions. We looked at the protein expression level of IL-1β to confirm the observed effects. Our results showed that CGA had a discernible impact on siBcl-2 and a suppressive effect on apoptosis. Additionally, we validated the expression of Bcl-2 in the co-delivery group using quantitative real-time PCR and immunofluorescence assay. These analyses consistently demonstrated a significant increase in Bcl-2 mRNA and protein levels compared to the control groups (Fig. 6D). This supports the hypthesis that the co-delivery of CGA and Bcl-2 targeted siBcl-2 effectively enhances chondrocytes’ survival.

## Discussion

Extracellular and intracellular signaling mechanisms regulate apoptosis and have unique physical and biochemical hallmarks. It has a variety of biological functions and is linked to degenerative disorders, autoimmune illnesses, and cancer [42]. This cell death process involves some pro- and antiapoptotic proteins that interact to balance the entire process of apoptosis [43]. It is well-documented that the pathogenesis of bone disorders is associated with chondrocyte apoptosis and abnormal leakage of extracellular matrix proteins [44]. Such conditions cause fundamental cell proliferation and survival abnormalities in decreasing antiapoptotic proteins such as B-cell lymphoma 2 (Bcl-2) [45]. Generally, B-cell lymphoma 2 (Bcl-2) is widely believed to be an essential apoptosis suppressor. However, the mechanism underlying its expression and functions in chondrocytes remains unknown. Thus, we analyzed Bcl-2 expression under different conditions to assess its impact on chondrocytes’ complementary proteins to overcome possible complications. The present study provides the evidence regarding a apoptotic cell death in Bcl-2 suppressed chondrocytes,  and its role in the expression of Bax/Bak mediated flux of Cytochrome C and executioner caspases (Caspase-3, Caspase-7) in the maturation of IL-1β. Both caspases are commonly believed to be responsible for coordinating the destruction phase of apoptosis by cleaving a wide variety of protein substrates. This is the case regardless of the particular stimulus that initially triggered cell death with the involvement of Bcl-2 [46]. Bruey et al. found that the relationship between Bcl-2 levels and Interleukin-1β inhibition is related to the reduction of NALP1 activation. NALP1 is a protein component of the inflammasome and is responsible for further activating caspase 1 [47]. Our findings support the hypothesis that Bcl-2 prevents chondrocytes from undergoing unusual cell death. In addition, combining siBcl-2 with antioxidants, i.e., Chlorogenic acid (CGA), induced significant apoptosis inhibition of chondrocytes. Such RNA interference utilization has led to the focus on siRNA-based therapeutics as the breakthrough for treating various diseases without any drawback [48]. Moreover, it has many advantages, including high biological activity, specificity, efficiency, and safety with low toxicity compared to the traditional treatment options [49]. Therefore, it has been subjected to expanding attention by researchers throughout the globe for treating different bone disorders in both humans and animals.

**Fig. 7:**
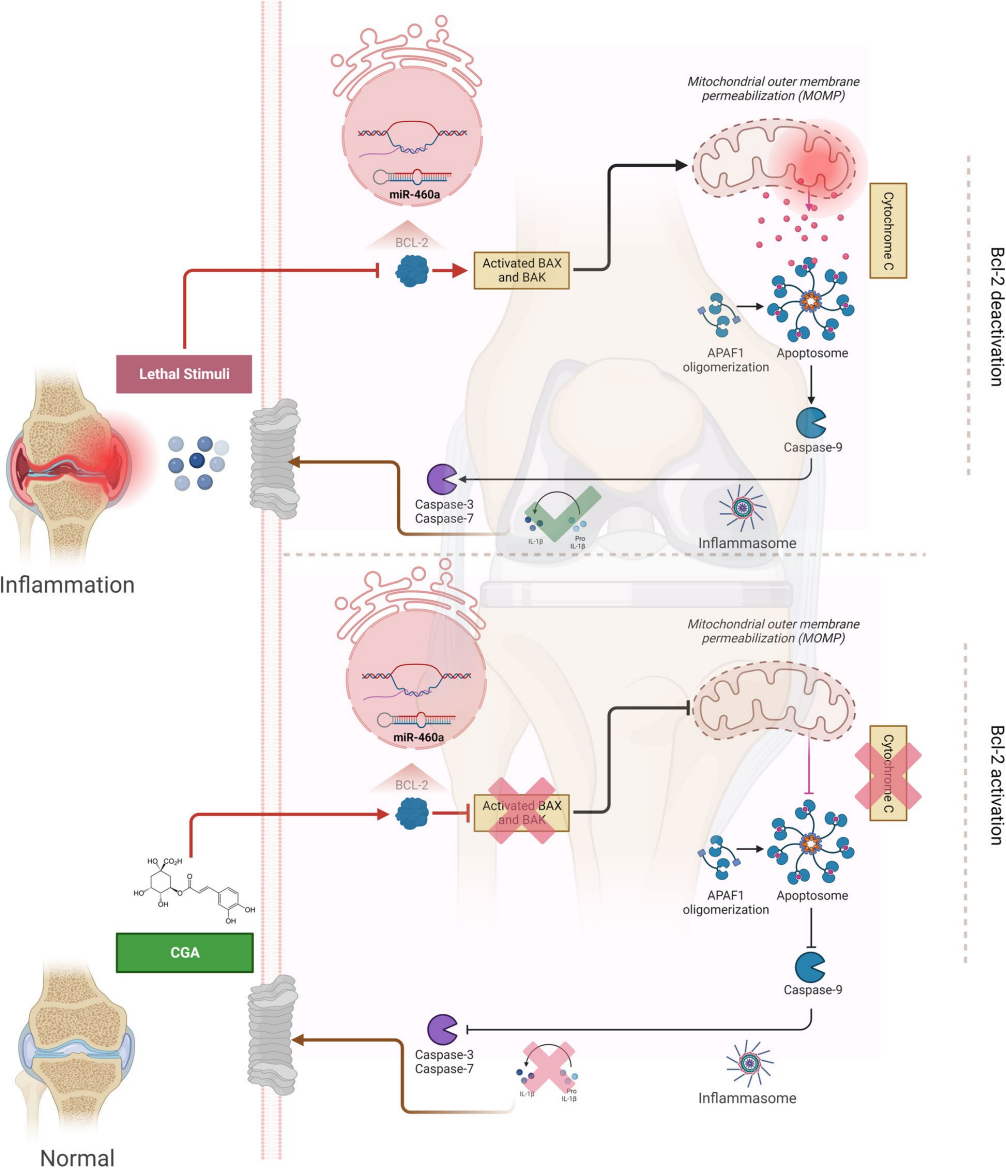
Mechanism insight in suppressing B-cell lymphoma 2 (Bcl-2) in chondrocytes, leading to apoptotic and inflammasome events and its role under the repercussion of CGA

Tibial dyschondroplasia (TD) is an important bone condition, causing a significant economic problem in the poultry industry [50]. Recent studies have suggested that the chondrocytes’ damage plays a crucial role in the development of TD due to their involvement in cartilage homeostasis and bone development [15]. The cartilage is a vital skeleton component, providing a smooth, lubricated surface for joint movement with the capability of absorbing mechanical force. The cartilage also plays a critical role in bone growth and development by serving as a template for bone formation during endochondral ossification [51]. Hence, chondrocyte apoptosis is a significant factor in most bone disorders. Normally, chondrocytes show higher proliferation but reduced apoptosis, which has been connected to an increase in the synthesis of antiapoptotic proteins [42]. Also, our current study found that suppression of Bcl-2 induces apoptosis in chondrocytes, further urging pro-apoptotic proteins (Bax, Bak) mediated Cyto C flux and interleukin-1β production. Moreover, the chondrocytes that underwent abnormal apoptosis are morphologically different, with less expression level of chondrogenic markers, i.e., COL1A2 and aggrecan. Any flaw in COLA12 would result in either qualitative or quantitative changes, depending on the location of the mutation [52]. Aggrecan is another important proteoglycan secreted by chondrocytes and is found in articular cartilage [53]. Thus, both macromolecules contribute to the formation of a “decellularized extracellular matrix” (dECM), which is directly related to the preservation of the normal environment to encourage cell proliferation and differentiation [54]. Therefore, decreasing these macromolecules may affect both normal and abnormal skeletal growth. According to some researchers, the higher level of caspases and IL-1β leads to the degradation of these chondrogenic markers [55, 56]. Parallel results were observed in our study, where pro-apoptotic proteins, i.e., Bax/Bak in Bcl-2 knockdown chondrocytes, were more transcribed with a low level of COL1A2 and aggrecan, which further alter the chondrocytes’ morphology. The reason is the involvement of apoptosis-independent pathways under the effect of executioner caspases [57]. Sharif et al. demonstrated that the expression of apoptosis-related mediators such as Caspase-3 (Casp3) is always higher in osteoarthritis cartilage compared to non-arthritic individuals. Also, the level of IL-1β is significantly increased in such cases [58]. Similarly, we found that knocking down Bcl-2 in chondrocytes induced a parallel inflammasome pathway, which in turn advanced the maturation of IL-1β even further. According to many researchers, Bcl-2 is a downstream target of microRNAs (miRNAs), resulting in trigger/ cellular death [59–62], as our previous findings also suggested its role under the miR-460a [63]. Moreover, recently discovered evidence supports these outcomes in macrophages regarding the role of Bcl-2 in inflammasome [64, 65]. Hence, the expression status of Bcl-2 is a key antiapoptotic protein in various disease conditions, including solid tumors and hematological malignancies. In numerous scenarios, such as cancer, suppressing Bcl-2 is vital to counteract apoptosis. As a result, scientists have been exploring the application of siBcl-2 therapeutics, utilizing small interfering RNA to target and inhibit Bcl-2 expression. However, despite the promising potential of this approach, certain limitations have impeded its effectiveness. Therefore, developing a standardized therapeutic strategy that can effectively regulate the expression of Bcl-2 becomes paramount, paving the way for improved treatment outcomes. In this study, we suggest using CGA, a therapeutic approach shown to regulate apoptosis, as part of a method for dispensing siRNA. Our theory is based on the idea that Chlorogenic acid and siRNA might work together to control how much Bcl-2 should be expressed. Through co-delivery experiments, we provide evidence supporting our hypothesis. The same findings were observed, where downregulating Bcl-2 expression with antisense oligonucleotides increased apoptosis and urged cancer cells to be more sensitive to chemotherapy drugs [66–68]. Even though their shared ability to specifically target the same messenger RNA (mRNA), the mechanisms by which small interfering RNA and antisense oligos suppress mRNA expression are distinctive [69]. The same findings were observed in our current study, where the siRNA and Chlorogenic acid (CGA) group collectively showed less apoptosis due to the greater sensitivity of chondrocytes toward CGA. The CGA is an excellent therapeutic agent in controlling unusual apoptotic events in our previous research [15, 26, 37]. According to our current findings, CGA attenuated Bcl-2 mediated siRNA’s apoptosis in chondrocytes by regulating the mitochondrial apoptotic pathway. In addition, it specifically regulated the chondrogenic proteins to exert a protective effect in chondrocytes. So, it might be a practical approach for suppressing further phenomenal signaling of Bax and Bak-mediated Cyto C flux with the blockage of the mitochondrial apoptosis pathway. The same findings have been reported by Dkhil et al. and Domitrovic et al., where CGA effectively prevented excessive apoptosis [70, 71]. Also, Ali et al. demonstrated that pre-treated rats with CGA significantly modulated the mRNA levels of apoptosis-related proteins [72]. Similarly, our previous findings from the same project found the involvement of CGA in deactivation of apoptosis and proinflammatory cytokines, e.g., interleukin-1β (IL-1β) [15, 26, 37]. But there is still a lot of speculation about Bcl-2 and how much of a role executioner caspase, i.e., (Casp3, Casp7) plays in cellular destruction. We thus investigated whether or not these caspases could process concurrently. Since the cleaved forms of both caspases decreased under CGA and increased under siRNA, we conducted an in vitro experiment via siBcl-2 and its counter with CGA, which resulted in significantly more efficiency for regulating both caspases. Under CGA pre-treatment, we detected a strong ability against such events. It implies that both are tightly connected and active during the destruction phase of apoptosis owing to the reduction in Bcl-2 protein levels [73]. Similar results were obtained by Walsh et al., who discovered that Casp3 and Casp7 were two completely different proteases that played non-overlapping but unique functions in the machinery responsible for cell death [74]. From all these perspectives, Bcl-2 was found to having connection with all suppression and overexpression of complementary proteins, e.g., Coll1A2, Aggrecan, Cyto C, Bax, Bak, executioner caspases, and IL-1β maturation. In summary, we showed for the first time that small interfering RNA (siRNA) against Bcl-2 exerts a provocative role in chondrocytes’ apoptosis, which participates in the pathogenesis of chondrocyte-related disorders. Moroever, our investigation delved into the significant role of CGA (Chlorogenic acid). We unearthed a promising therapeutic target for conditions related to bone disorders, where using siRNA-based therapeutics is essential. These findings shed light on the potential of CGA as a viable candidate for targeted therapies, opening up new possibilities for using siRNA-based therapeutics (Fig. 7).

As far as the clinical usage of siBcl-2 and CGA is concerned, their combination could be used during all pathological conditions of bone where chondrocytes' balanced apoptosis is concerned, like tibial dyschondroplasia, osteoarthritis, rheumatoid arthritis, and all other chondrocyte-related disorders.

There are also some limitations, e.g., contradictory results have been found in the few types of research that have looked at the effects of silencing a single gene over the whole genome. But, still, it might be a discovery in chondrocytes that needs further study about using different siRNAs with any other therapeutic drug in controlling complementary pathways, aiming to find a better option with fewer complications. While our investigation gives vital insights into the function of Bcl-2 in chondrocyte apoptosis and inflammasome activation, we acknowledge several limitations. Firstly, siRNA knockdown may not always reach 100% effectiveness, and leftover Bcl-2 expression might impact the results. Additionally, apoptotic assays might demonstrate variability owing to several variables, including cell culture conditions and assay specificity.

## Conclusions

Our findings provide compelling evidence for the critical role of Bcl-2 in upholding apoptosis and inflammasome activation in chondrocytes. Manipulating Bcl-2 expression levels resulted in distinct cellular responses, shedding light on the regulatory mechanisms involved in chondrocyte function. Furthermore, co-delivery of Bcl-2 with CGA demonstrated potential antagonistic effects, suggesting a promising avenue for therapeutic interventions. Understanding the intricate molecular pathways governing chondrocyte behavior is crucial for addressing cartilage-related conditions. Therefore, the current study contributes to our knowledge of the underlying mechanisms controlling apoptosis and inflammasome activation in chondrocytes for promoting cartilage health and combating chondrocyte-associated disorders.

### Supplementary Information

**Additional file 1: Supplementary Fig. 1.** The research layout of in vitro experiment for investigating Bcl-2 role in Apoptosis and inflammasome activation. **Supplementary Table 1.** Primers used in this study. **Supplementary Table 2.** Sequences for siRNA-BCL2.

## Abbreviations

Bcl-2: b-cell lymphoma-2
bp: bandpass
GP: growth plate
miRNA: microRNA
miR-460a: gga-miR-460a-5p
IL-1β: interleukin-1β
