# Supplementary material for: Modulation of apoptosis and Inflammasome activation in chondrocytes: co-regulatory role of Chlorogenic acid
Source: Cell Commun Signal. 2024 Jan 2;22:2. doi: 10.1186/s12964-023-01377-w (PMC10759508; doi:10.1186/s12964-023-01377-w)
Supplement: Supplementary file 2 — Additional file 1: Supplementary Fig. 1. The research layout of in vitro experiment for investigating Bcl-2 role in Apoptosis and inflammasome activation. Supplementary Table 1. Primers used in this study. Supplementary Table 2. Sequences for siRNA-BCL2. [file 12964_2023_1377_MOESM1_ESM.docx]

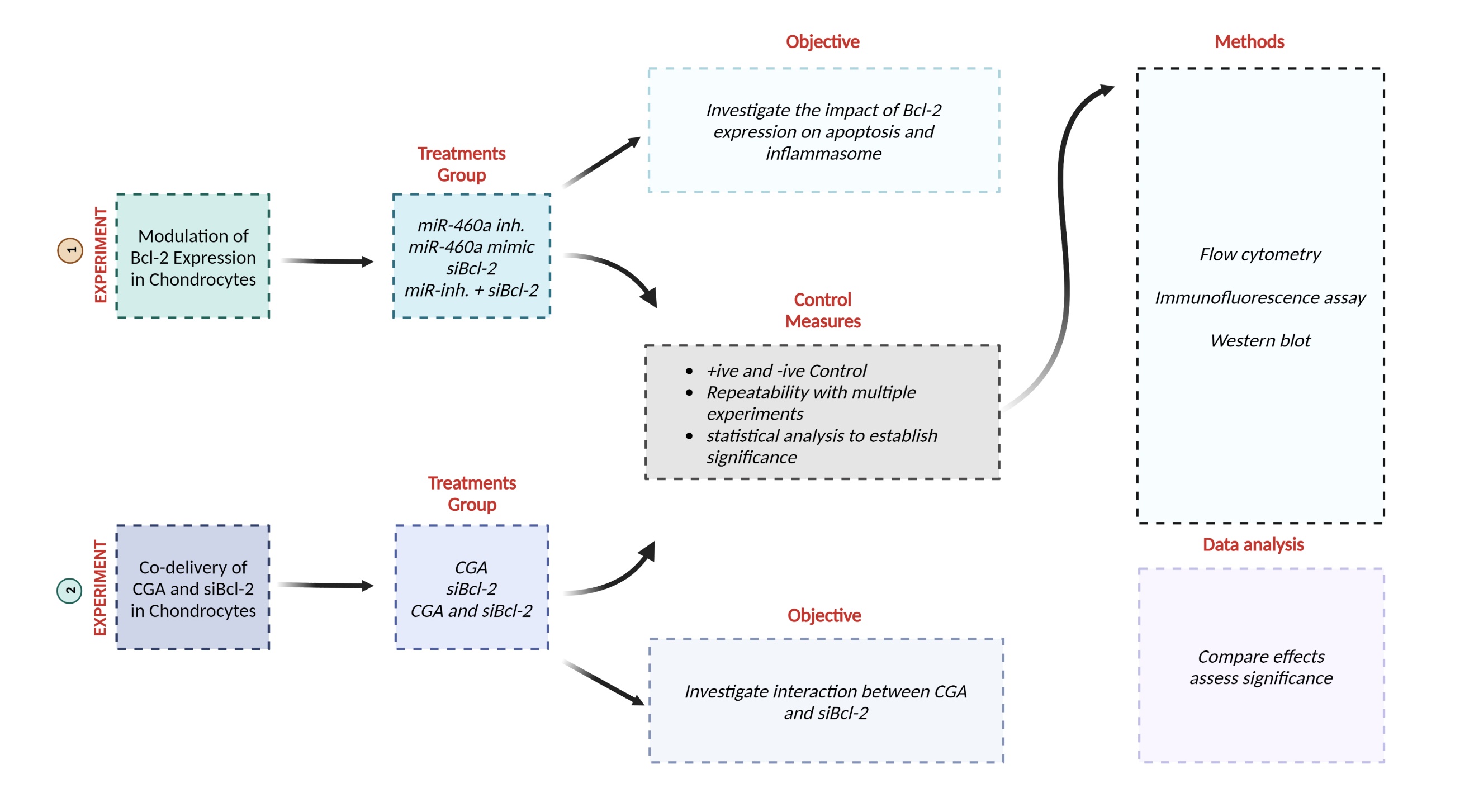


**Supplementary Figure. 1** The research layout of in vitro experiment for investigating Bcl-2 role in Apoptosis and inflammasome activation.

**Supplementary Table 1:** Primers used in this study.

| **Gene** | **Forward Primer Sequence (5′ to 3′)** | **Reverse Primer Sequence (5′ to 3′)** |
| --- | --- | --- |
| **miR-460a** | GTATACTTAGAAATGCAAATT | GTGCAGGGTCCGAGGT |
| **Collagen** | GCCCTTCTCACGACCACCAT | GGCTCCGTTGACCTCCCATA |
| **Aggrecan** | CTGGCTGCAAGAGAGACC | GCCCTCCTATTTCCCCCT |
| **Bcl-2** | GCAGGCAGCTTGAAAGAAAC | GCTGGCCTTTCATGACTCTC |
| **Bcl.xL** | CATCGTGGCTTTCTTCTCCTT | CAGCGTTGTTCCCATACAGA |
| **Cyto C** | TTCCCAGTGCCATACGGTTG | GCTTGTCCTGTTTTGCGTCC |
| **Bax** | GTGATGGCATGGGACATAGCTC | TGGCGTAGACCTTGCGGATAA |
| **Bak** | ACCCGGAGATCATGGAGA | GATGCCTTGCTGGTAGACG |
| **Caspase-3** | TGGCGATGAAGGACTCTTCT | TCATCTGGTCCACTGTCTGC |
| **Caspase-7** | CTGGCTGTTTCTCTGCCC | GTGGATGGAACGCACTGG |
| **NLRP3** | GGTTCCCTTCCCCTCACT | CTCGGGTAGGCTGGAGTT |
| **IL-1β** | CACACGAAGCTCGGACAC | GAGATGGCGTTCGTTCCC |
| **GAPDH** | GCCCAGAACATCATCCCA | CGGCAGGTCAGGTCAACA |
| **U6** | AAGTGTGTGAGGAGAGGCCC | CCGGGGGCGCCACAT |

**Supplementary Table. 2** Sequences for siRNA-BCL2

| siRNA-BCL2 | Sense | 5’- GGAGAAGAGGCUACGACAATT-3’ |
| --- | --- | --- |
|  | Antisense | 5’- UUGUCGUAGCCUCUUCUCCTT-3’ |
| siRNA-NC | Sense | 5’- UUCUCCGAACGUGUCACGUTT-3’ |
|  | Antisense | 5’- ACGUGACACGUUCGGAGAATT-3’ |
